# Mr. Jones's Case of Twins

**Published:** 1811-04

**Authors:** John Jones

**Affiliations:** Member of the Royal College of Surgeons, London. Guisbro' Yorkshire


*To the Editors of the Medical and Physical Journal.*

Gentlemen,

THE following singular case of twins having lately occurred in this neighbourhood, I am induced to request you will insert it in your highly useful periodical work, that others may have an opportunity to pass their opinion on a case, which I consider of the highest practical importance. A woman between thirty and forty years of age, and rather corpulent, was delivered on Sunday, the 10th of February; nothing particular occurred during her labour, except that the child was smaller than common. The accoucheur, probably from the rare occurrence of twins, did not examine sufficiently to discover another fœtus. On the Friday following, the woman declared to him that she still felt the motion of a child; in consequence of which he examined *per vaginam*, and could distinctly feel the head of a fœtus situated very high in utero. The os uteri was contracted to about the size of half-a-crown. The Sunday following he requested me to see his patient. On applying my hand upon the abdomen I was immediately convinced of the existence of another child; the motion of which I felt distinctly. The woman having continued as well as could be expected since her former delivery, I gave my opinion, decidedly, to wait patiently, leave the result to nature, and cautiously to observe her operations, which perfectly coincided with the sentiments of the practitioner employed. On Sunday, the 24th of February, a fortnight from the birth of her first child, labour recommenced, and she was soon delivered of a fine boy, much larger than the former. The mother is doing perfectly well, not having felt any unfavourable symptoms since the commencement of her confinement. Her milk did not appear till two days after the birth of the second child.

From the circumstance of the above case terminating so favourably, and having *heard* of other similar cases, particularly one, in which six weeks had elapsed, and another, a fortnight a fortnight between the birth of each child; I am almost inclined to pay some deference to the rejected doctrine of superfœtation; or at least, to doubt the propriety of the present established practice, to deliver *per artem*, if nature is tardy in her efforts to expel the second child. Most of the public teachers of the present day are not, in my opinion, sufficiently explicit in their directions for the management of cases similar to the one I have related; namely, *where the placentæ are separate in the uterus, and each follows the body to which it belongs*. Dr. Hamilton certainly gives directions rather more fully: he says, "the placentæ of twins are often distinct from each other in the uterus, and so loosely connected to it, that one may entirely separate before the second child be born, so that labour pains will sometimes cease for two or three days, and there is the same interval between the birth of the children." A little further, he says, "in this interval the patient is apt to suffer from impatience, and anxiety, floodings frequently come on and the labour is more painful and hazardous, in proportion as the time of delivery is protracted." It is with the utmost deference that I differ from such high authority; but I would ask you and other professional gentlemen, if we are not justified in drawing the following inference from the cases I have mentioned; that it would be more prudent, *if no unfavourable symptoms occur*, to leave the expulsion of the second child to nature, rather than to interfere according to the present practice; the objection that there is danger from hæmorrhage ensuing, in my opinion, principally exists where the placentæ have been at all connected; and that if they are perfectly distinct, there is little more risk from that cause than in common natural labour; as I conceive that the uterus would be so much relieved from the expulsion of one child, as to be able to contract sufficiently to guard against such an accident; but even should hæmorrhage be the consequence, yet, would it not occur soon after delivery of the first child? and consequently would sufficiently justify the interference of art. I am much inclined to suppose, that instead of the hazard being increased in proportion as the time of delivery is protracted, as Dr. Hamilton asserts, it is considerably diminished; at least, such an inference I think we are justified in drawing from the case I have related.

I am Gentlemen,

*Guisbro' Yorkshire,* Yours, &c.

*March* 8, 1811. JOHN JONES,*

Member of the Royal College of Surgeons, London.

---

* We hope in a future number, probably the next, to give the opinion

### *To the Editors of the Medical and Physical Journal.*

GENTLEMEN, nion and observations of a London professor, on this interesting subject. At present we are disposed to adopt, as a general principle, the practice recommended by our ingenious correspondent, liable, however, to exceptions and limitations, arising from the peculiarities of individual cases. If a second child is discovered in the uterus immediately on the delivery of the first, enveloped in its proper membranes, and having a distinct placenta, even if no hæmorrhage to a dangerous degree exist, and there be a total absence of uterine action, it may be a question, if in this open and practicable state of the parts it be proper to rupture the membranes of the second child, and deliver immediately. But if so much time has elapsed before the second child is discovered as shall have given the uterus leisure to contract; and if the os uteri, as in Mr. Jones's case, has diminished to the size of a half-crown, there existing neither dangerous hæmorrhage or other alarming symptom, what reason can be urged for proceeding to immediate delivery *per artem?* The anxiety and solicitude that the mother will feel from the lingering prospect of a second delivery, and the labour becoming more painful and hazardous in proportion as it is protracted, according to Prof. Hamilton, do present some motive, under the most favourable existing circumstances, for immediate delivery of the second child. Will it be difficult to decide between this remote hazard, and the direct danger arising from a manual dilatation of the os uteri; an operation, which if not only, is best justified by the existence of an hæmorrhage that puts the patient's life in danger? The present rational state of the *ars obstetrica*, and the high degree of science manifested by its professors, cannot suffer this question long to remain in doubt. EDITOR.

